# Effects of basal and premixed insulin on glycemic control in type 2 diabetes patients based on multicenter prospective real‐world data

**DOI:** 10.1111/1753-0407.13245

**Published:** 2022-01-13

**Authors:** Ying Peng, Peihong Xu, Juan Shi, Yifei Zhang, Shujie Wang, Qidong Zheng, Yufan Wang, Tingyu Ke, Li Li, Dong Zhao, Yuancheng Dai, Qijuan Dong, Bangqun Ji, Fengmei Xu, Weiqiong Gu, Weiqing Wang

**Affiliations:** ^1^ Department of Endocrine and Metabolic Diseases, Shanghai Institute of Endocrine and Metabolic Diseases, Ruijin Hospital Shanghai Jiao Tong University School of Medicine Shanghai China; ^2^ Shanghai National Clinical Research Center for Metabolic Diseases, Key Laboratory for Endocrine and Metabolic Diseases of the National Health Commission of the PR China, Shanghai Key Laboratory for Endocrine Tumor, State Key Laboratory of Medical Genomics, Ruijin Hospital Shanghai Jiao Tong University School of Medicine Shanghai China; ^3^ Department of Pharmacy, Ruijin Hospital Shanghai Jiao Tong University School of Medicine Shanghai China; ^4^ Department of Internal Medicine The Second People’s Hospital of Yuhuan Yuhuan China; ^5^ Department of Endocrinology and Metabolism, Shanghai General Hospital Shanghai Jiao Tong University School of Medicine Shanghai China; ^6^ Department of Endocrinology The Second Affiliated Hospital of Kunming Medical University Kunming China; ^7^ Department of Endocrinology Ningbo First Hospital Ningbo China; ^8^ Center for Endocrine Metabolism and Immune Diseases, Beijing Luhe Hospital Capital Medical University Beijing China; ^9^ Department of Internal Medicine of Traditional Chinese Medicine Sheyang Diabetes Hospital Yancheng China; ^10^ Department of Endocrinology and Metabolism People’s Hospital of Zhengzhou Affiliated Henan University of Chinese Medicine Zhengzhou China; ^11^ Department of Endocrinology Xingyi People’s Hospital Xinyi China; ^12^ Department of Endocrinology and Metabolism, Hebi Coal (Group), Ltd General Hospital Hebi China

**Keywords:** basal insulin, body mass index, glycemic hemoglobin, premixed insulin, type 2 diabetes, 型糖尿病, 预混胰岛素, 基础胰岛素, 糖化血红蛋白, 体重指数

## Abstract

**Background:**

To investigate the different efficacies of glycemic control between basal and premixed insulin in participants with type 2 diabetes (T2DM) when non‐insulin medications fail to reach treatment targets.

**Methods:**

This was a prospective, large‐scale, real‐world study at 10 diabetes centers in China. Between June 2017 and June 2021, we enrolled 1104 T2DM participants initiated with either once‐daily basal insulin or twice‐daily premixed insulin when the glycosylated hemoglobin (HbA1c) control target was not met after at least two non‐insulin agents were administered. A Cox proportional hazards regression model adjusting for multiple influencing factors was performed to compare the different effects of basal and premixed insulin on reaching the HbA1c control target.

**Results:**

At baseline, basal insulin (57.3%) was prescribed more frequently than premixed insulin (42.7%). Patients with a higher body mass index (BMI) or higher HbA1c levels were more likely to receive premixed insulin than basal insulin (both *p* < 0.001). After a median follow‐up of 12.0 months, compared to those with premixed insulin, the hazard ratio for reaching the HbA1c target to those with basal insulin was 1.10 (95% CI, 0.92‐1.31; *p* = 0.29) after adjustment, and less weight gain was observed in those with basal insulin than with premixed insulin (percentage change of BMI from baseline −0.37[5.50]% vs 3.40[6.73]%, *p* < 0.0001).

**Conclusions:**

In this real‐world study, once‐daily basal insulin was more frequently prescribed and had similar glycemic control effects but less weight gain compared with twice‐daily premixed insulin when used as initiation therapy for those in whom glycemic control with non‐insulin medications failed.

## Graphical abstract

**Highlights**

- In real‐world practice in China, once‐daily basal insulin was prescribed more often than twice‐daily premixed insulin to type 2 diabetes patients uncontrolled with non‐insulin medications.
- Treatment with once‐daily basal insulin showed similar effects on reaching glycosylated hemoglobin targets compared with twice‐daily premixed insulin, even after stratification by multiple influencing factors. Less weight gain was observed in those treated with basal insulin than in those treated with premixed insulin, which might be at least partially attributed to the lower insulin dosage administered in the former group.

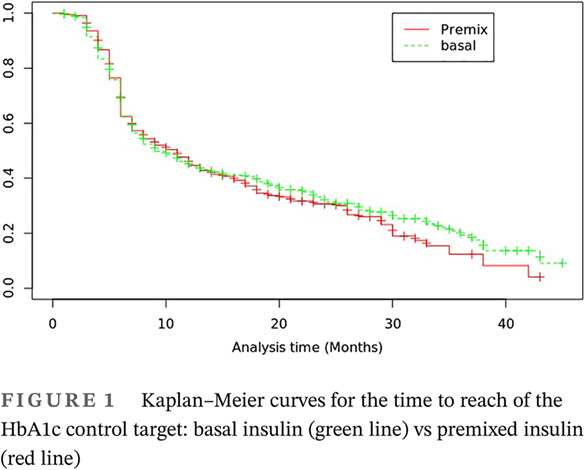

## 摘要

**背景:**

探究当非胰岛素降糖药物不能达到控制目标时, 基础胰岛素和预混胰岛素对2型糖尿病(T2DM)患者血糖控制的疗效差异。

**方法:**

这是一项在中国10个糖尿病中心进行的前瞻性大规模真实世界研究。在2017年6月至2021年6月期间, 我们共纳入了1104位2型糖尿病患者, 均在使用至少两种非胰岛素降糖药治疗时糖化血红蛋白(HbA1c)未达标后开始启用每天一次基础胰岛素或每天两次预混胰岛素治疗。采用Cox比例风险回归模型矫正多个影响因素, 比较基础胰岛素和预混合胰岛素对HbA1c控制的效果。

**结果:**

基线数据显示, 基础胰岛素的处方频率(57.3%)高于预混胰岛素(42.7%)。体重指数(BMI)或HbA1c水平较高的患者接受预混胰岛素治疗的可能性高于基础胰岛素(P<0.001)。经过中位12个月的随访, 与预混胰岛素组相比, 校正后的基础胰岛素组患者HbA1c达标的风险比(95%CI)为1.10(0.92‐1.31, P=0.29), 而体重增加的程度相对较小(BMI增加百分比为‐0.37[5.50]% vs. 3.40[6.73]%, P<0.0001)。

**结论:**

在这项真实世界研究中, 在使用非胰岛素降糖药物血糖控制失败后作为起始治疗时, 每日一次的基础胰岛素与每日两次预混胰岛素治疗方案相比, 前者更为常用。两者虽具有相似的血糖控制效果, 但每日一次的基础胰岛素治疗体重增加更少。

## 1 INTRODUCTION

The prevalence of diabetes has increased dramatically in the past three decades. Together with its related acute and chronic complications, diabetes has become a worldwide public health problem that accounts for nearly 12% of overall health care costs globally.^1^, ^2^ In China, the situation is worse.^3^, ^4^, ^5^, ^6^ According to a recent survey, approximately 12.8% of adults living in China suffer from diabetes, leading China to have the largest absolute number of diabetes patients worldwide.^6^ Moreover, despite substantial advances in treatment and care, a high proportion of patients with diabetes still cannot reach treatment targets.^3^, ^7^ Currently, when glycosylated hemoglobin (HbA1c) is not well controlled, add‐on therapy with insulin is the next step for patients with type 2 diabetes (T2DM) who fail glycemic control with lifestyle intervention and non‐insulin agents, as recommended by most of the treatment guidelines.^8^, ^9^, ^10^, ^11^, ^12^ However, which pattern should be the first choice of insulin regimen at this stage is not consistent across countries. Chinese guidelines for the management of T2DM recommend the initiation of insulin therapy with either basal insulin once daily or premixed insulin twice daily when glycemic targets could not be met after non‐insulin medications;^9^ however, basal insulin is the only recommendation at this step by the American Diabetes Association (ADA) and the European Association for the Study of Diabetes (EASD) statements.^8^, ^10^ This difference is mainly based on the consideration of the special diet structure (high percentage of carbohydrates) and etiologic characteristics of T2DM patients in China.^13^ However, the different effects of the two regimens in clinical use had not been fully investigated. Currently, limited evidence has been obtained in a few investigations.^14^, ^15^, ^16^, ^17^, ^18^ For instance, one cross‐sectional survey of 602 Chinese hospitals demonstrated that premixed insulin showed better control of HbA1c than basal insulin for T2DM patients.^14^ Another pragmatic study with a randomized controlled design indicated a similar change in least squares mean HbA1c after 24 weeks of treatment between the basal and premixed insulin groups.^15^ However, currently it is still unclear which treatment modality is preferred by doctors and is more effective for patients in China in real‐world application.

Therefore, we conducted a prospective study with up to 47 months of follow‐up of T2DM patients to investigate the different characteristics of basal and premixed insulin use in real‐world practice at 10 diabetes centers in China. The main aims of the current study were to investigate (1) the habits of Chinese endocrinologists in the choice of insulin regimens when T2DM patients failed with non‐insulin medications and to evaluate the potential influencing factors, (2) different efficacies on glycemic control between basal and premixed insulin used in these patients, and (3) stratification analyses with multiple influencing factors to evaluate the different efficacies of each insulin regimen in the subgroups.

## 2 METHODS

### 2.1 Study population and design

From June 2017 through June 2021, we enrolled T2DM participants from 10 diabetes centers known as National Metabolic Management Centers (MMCs), which were located in 7 provinces in China, with follow‐up data collected until September 2021. A detailed introduction of the pilot, nationwide, prospective MMC program can be found in previous publications (ClinicalTrials.gov number, NCT03811470).^5^, ^19^, ^20^ In the present study, we enrolled eligible participants from these MMCs according to the following criteria: (1) T2DM participants aged ≥18 years who were treated with lifestyle intervention and at least two kinds of non‐insulin medications at baseline; (2) participants with unmet glycemic control targets (defined as HbA1c ≥ 7%) for at least 3 months who were switched to either once‐daily basal insulin or twice‐daily premixed insulin as the initiating insulin regimen pattern; and (3) participants who attended a follow‐up visit for at least 3 months, during which period the insulin administration pattern should not be changed regardless of the changes in insulin dosage.

The Ethics Committees at Ruijin Hospital, Shanghai Jiao Tong University School of Medicine, and at the other participating centers (subsequently if necessary) approved the study protocol. This study abided by the provisions of the Declaration of Helsinki, and written informed consent was provided by all the participants.

### 2.2 Data collection and treatment

At baseline, a comprehensive clinical evaluation of each participant at the 10 MMCs, including a detailed questionnaire, anthropometric parameters, laboratory testing data, and diabetes‐related complication examinations, was carried out and updated at each visit when necessary, according to a standard operation procedure.^5^, ^19^ Briefly, participants were in light clothes with shoes off to measure height and body weight to calculate the body mass index (BMI). Blood pressure was tested after ≥5 minutes of rest in the seated position. Blood tests including fasting blood glucose (FBG), fasting serum C peptide, HbA1c, and serum lipid profiles tested at each local center were collected and reported in the present analysis.

During baseline and each follow‐up visit, the investigators at each center prescribed the proper treatment pattern to the participant and adjusted the insulin dosages and concomitant non‐insulin medications based on their own judgment and according to the regular treatment guideline at that time without special restriction. The type, dosage, use frequency, and duration of each medication, as well as the corresponding changes, were recorded in the digital database system. Basal insulin referred to the long‐acting forms of insulin, including insulin glargine, detemir, and degludec, in the present study. Since very few participants (n = 6) met the inclusion criteria and used once‐daily intermediate‐acting insulin (neutral protamine Hagedorn), we did not include this type of insulin as a basal insulin regimen in the final analysis. The premixed insulin included a different percentage of mixture of a short/fast‐acting insulin with an intermediate/long‐acting insulin. The hypoglycemic episodes during follow‐up visits were also recorded.

The primary outcome was the time to reach the HbA1c control target (defined as HbA1c < 7% or percentage decrease of HbA1c from baseline >10%). The secondary outcomes included the differences and changes in other metabolic parameters from baseline to the end of follow‐up between the two groups.

### 2.3 Statistical analysis

All analyses were performed using R software (version 4.0.5, R Foundation for Statistical Computing). We considered a two‐sided *p* value < 0.05 to be statistically significant. Continuous variables are presented as mean ± SD for normally distributed variables or the median (interquartile range, IQR) for skewed variables. Categorical variables are presented as numbers and proportions. The analyses of continuous and categorical variables to assess differences among the two treatment patterns were determined by one‐way analysis of variance or the *χ* ^2^ test. The change of metabolic parameters after treatment within each group was analyzed using the paired sample *t* test. For the primary outcome, a Cox proportional hazards regression model was performed to compare the different effects of basal and premixed insulin, including univariable and multivariable adjustments, on variables including sex, age, duration of diabetes, total cholesterol, fasting serum C peptide, systolic blood pressure (SBP), and HbA1c values at baseline. In addition, we performed subgroup analyses in prespecified subgroups of age, sex, HbA1c, BMI, SBP, and total cholesterol using multivariable Cox proportional hazards regression models with full adjustment. For the percentage change in other metabolic parameters from baseline, an analysis of covariance model was used to examine the difference between basal and premixed insulin treatment groups adjusted for sex, age, duration of diabetes, total cholesterol, fasting serum C peptide, SBP at baseline, and corresponding baseline value. While comparing the percentage change of the diastolic blood pressure (DBP) and low‐density lipoprotein (LDL) cholesterol levels between groups, baseline SBP and total cholesterol were not adjusted accordingly.

## 3 RESULTS

### 3.1 Baseline clinical characteristics of the participants

In total, 1104 T2DM participants with a mean (SD) age of 57.65 (10.21) years at the 10 study centers were enrolled in the present analysis, among whom 471 (42.7%) participants received twice‐daily premixed insulin and 633 (57.3%) received once‐daily basal insulin. At baseline, clinical characteristics, including age, sex, diabetes duration, history of hypertension and dyslipidemia, and smoking and drinking histories, were not significantly different between the two groups (Table 1). However, compared with participants in the basal insulin group, those in the premixed insulin group had relatively higher FBG, HbA1c, BMI, waist circumference, blood pressure, and total and LDL cholesterol concentrations (all *p* < 0.01).

**TABLE 1:** Baseline characteristics of the participants

	Total	Premixed insulin	Basal insulin	*p* value
No. of participants	1104	471	633	
Male sex, n (%)	561 (50.82%)	227 (48.20%)	334 (52.76%)	0.150
Family history of diabetes, n (%)	638 (60.59%)	255 (56.92%)	383 (63.31%)	0.042
History of hypertension, n (%)	515 (46.90%)	232 (49.47%)	283 (44.99%)	0.159
Hypertensive medication use, n (%)	505 (45.83%)	227 (48.20%)	278 (44.06%)	0.193
History of dyslipidemia, n (%)	344 (31.39%)	144 (30.84%)	200 (31.80%)	0.785
Ideal smoking, n (%)	839 (76.27%)	359 (76.38%)	480 (76.19%)	0.998
Drinker, n (%)	100 (9.08%)	40 (8.51%)	60 (9.51%)	0.643
Age, y	57.65 ± 10.21	57.65 ± 9.93	57.65 ± 10.41	0.992
Duration of diabetes, y	10.68 ± 6.44	10.41 ± 6.54	10.89 ± 6.35	0.223
BMI, kg/m^2^	25.27 ± 3.47	25.71 ± 3.49	24.95 ± 3.42	0.0003
Body weight, kg	67.07 ± 11.73	67.77 ± 11.76	66.54 ± 11.70	0.089
Visceral fat area, cm^2^	94.57 ± 40.82	93.85 ± 39.63	95.21 ± 41.90	0.641
Waist circumference, cm	90.24 ± 9.78	91.21 ± 10.03	89.48 ± 9.53	0.005
SBP, mmHg	131.54 ± 18.93	133.97 ± 20.42	129.73 ± 17.55	0.0003
DBP, mmHg	76.13 ± 11.37	77.80 ± 12.01	74.88 ± 10.70	<0.0001
Fasting blood glucose, mmol/L	11.26 ± 4.00	12.10 ± 4.58	10.63 ± 3.36	<0.0001
Fasting serum C peptide, ng/mL	1.87 (1.26, 2.69)	1.90 (1.24, 2.80)	1.82 (1.30, 2.58)	0.751
HbA1c, %	9.49 ± 1.67	9.99 ± 1.78	9.12 ± 1.48	<0.0001
Triglycerides, mmol/L	1.59 (1.08, 2.36)	1.63 (1.14, 2.42)	1.55 (1.05, 2.30)	0.265
Total cholesterol, mmol/L	4.81 ± 1.31	4.96 ± 1.29	4.70 ± 1.32	0.001
HDL cholesterol, mmol/L	1.23 ± 0.36	1.25 ± 0.38	1.21 ± 0.34	0.106
LDL cholesterol, mmol/L	2.86 ± 1.04	3.06 ± 1.09	2.71 ± 0.98	<0.0001

*Note*: Data are mean ± SD, median (interquartile range), or n (%).

*Note*: The *p* values refer to comparison between premixed insulin group and basal insulin group using one‐way ANOVA or *χ* ^2^ test.

Abbreviations: ANOVA, analysis of variance; BMI, body mass index; DBP, diastolic blood pressure; HbA1c, glycosylated hemoglobin; HDL, high‐density lipoprotein; LDL, low‐density lipoprotein; SBP, systolic blood pressure.

### 3.2 Glycemic control

After a median (IQR) follow‐up of 12.0 (6.0, 23.0) months, the mean (SD) HbA1c values decreased significantly from 9.99 (1.78)% to 8.41 (1.73)% in the premixed insulin group, and from 9.12 (1.48)% to 7.99 (1.45)% in the basal insulin group (both *p* < 0.001).

**FIGURE 1:**
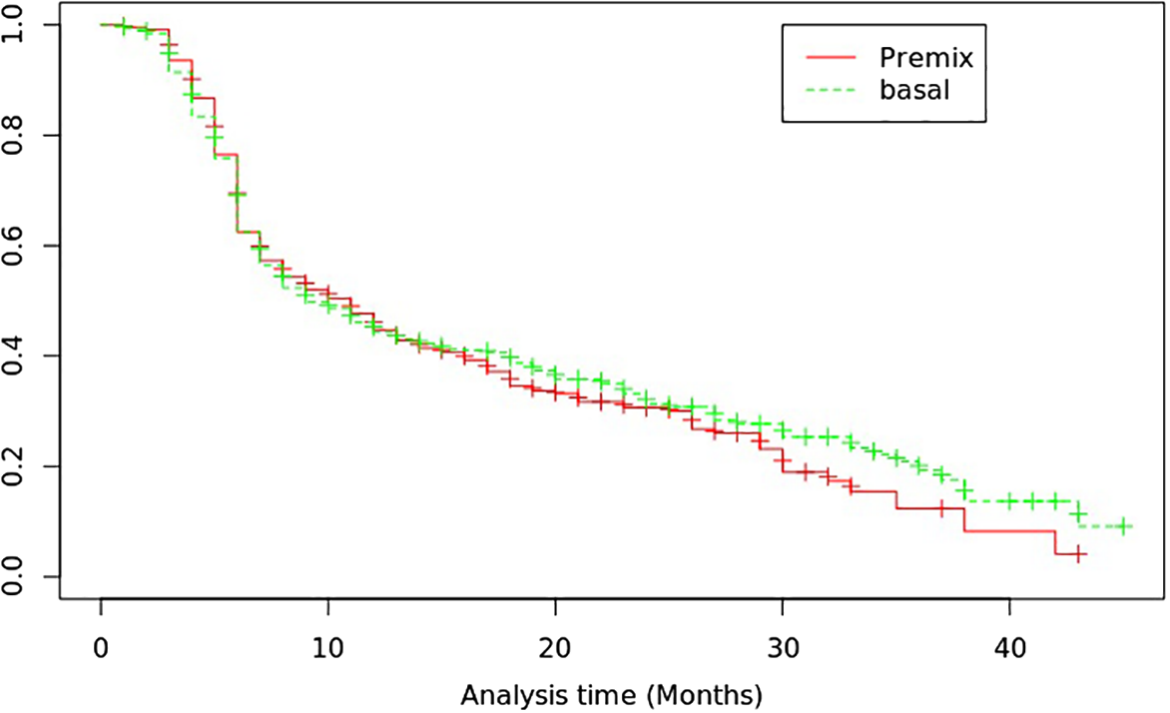
Kaplan–Meier curves for the time to reach of the HbA1c control target: basal insulin (green line) vs premixed insulin (red line)

After treatment, a total of 295 (62.6%) participants in the premixed insulin group and 361 (57.0%) participants in the basal insulin group achieved a glycemic control target (HbA1c <7% or percentage decrease of HbA1c value >10%). Compared with those treated with premixed insulin, the hazard ratio (HR) for the treatment target for those with basal insulin was 1.10 (95% CI, 0.92‐1.31; *p* = 0.29) after adjusting for multiple influencing factors (Figure 1, Table 2). We further analyzed the difference between basal and premixed insulin treatment groups in percentage HbA1c change from baseline to the last visit for multivariable adjustments (Table 3). The results indicated that no significant difference was found between the premixed and basal insulin group in mean (SD) percentage change of HbA1c (−13.30 [20.59]% vs −10.75 [17.69]%, *p* = 0.054).

**TABLE 2:** Hazard ratios for the treatment target of basal insulin and premixed insulin

	Treatment target	Unadjusted model		Adjusted model^a^	
	n (%)	HR (95% CI)	*p* value	HR (95% CI)	*p* value
Premixed insulin	295 (62.6%)	Reference		Reference	
Basal insulin	361 (57.0%)	0.96 (0.83, 1.13)	0.65	1.10 (0.92, 1.31)	0.29

*Note:* HR and 95% CI were evaluated using Cox proportional hazards regression model. Abbreviations: HbA1c, glycosylated hemoglobin; HR, hazard ratio.

^a^ Adjusted for sex, age, duration of diabetes, total cholesterol, fasting serum C peptide, systolic blood pressure, and HbA1c at baseline.

**TABLE 3:** Main clinical characteristics after treatments

	Total (N = 1104)		Premixed insulin (n = 471)		Basal insulin (n = 633)		
	Last visit	Percentage change (%)^a^	Last visit	Percentage change (%)^a^	Last visit	Percentage change (%)^a^	*p* value
BMI, kg/m^2^	25.55 ± 3.52	1.25 ± 6.34	26.59 ± 3.60	3.40 ± 6.73	24.76 ± 3.25	−0.37 ± 5.50	<0.0001
HbA1c, %	8.17 ± 1.58	−11.82 ± 18.99	8.41 ± 1.73	−13.30 ± 20.59	7.99 ± 1.45	−10.75 ± 17.69	0.054
Fasting blood glucose, mmol/L	9.23 ± 3.77	−10.47 ± 44.08	10.18 ± 4.19	−5.99 ± 50.56	8.53 ± 3.25	−13.81 ± 38.25	<0.0001
SBP, mmHg	131.86 ± 19.39	1.23 ± 15.16	136.57 ± 20.74	2.96 ± 16.48	128.28 ± 17.49	−0.07 ± 13.97	<0.0001
DBP, mmHg	75.29 ± 10.60	−0.22 ± 14.42	77.66 ± 11.24	0.76 ± 14.67	73.49 ± 9.73	−0.96 ± 14.19	0.0002
Total cholesterol, mmol/L	4.51 ± 1.17	−3.00 ± 24.92	4.70 ± 1.19	−3.18 ± 23.12	4.38 ± 1.14	−2.87 ± 26.14	0.025
Triglycerides, mmol/L	1.47 (0.99, 2.19)	3.74 ± 62.30	1.59 (1.07, 2.32)	11.53 ± 73.83	1.37 (0.93, 2.14)	−1.79 ± 51.98	0.0005
LDL cholesterol, mmol/L	2.55 ± 0.95	−4.85 ± 37.01	2.71 ± 0.96	−6.67 ± 32.20	2.43 ± 0.93	−3.55 ± 40.06	0.080

*Note*: The *p* values refer to comparison between premixed insulin group and basal insulin group using analysis of covariance model adjusted for sex, age, duration of diabetes, total cholesterol, fasting serum C peptide, SBP at baseline, and corresponding baseline value. While calculating the *p* values for DBP and LDL cholesterol percentage change between groups, baseline SBP and total cholesterol were not adjusted, respectively.

Abbreviations: BMI, body mass index; DBP, diastolic blood pressure; HbA1c, glycosylated hemoglobin; LDL, low‐density lipoprotein; SBP, systolic blood pressure.

^a^ The values are the changes from baseline divided by the baseline value.

The types of hypoglycemic medications analyzed in the present study are listed in the Table S1. The mean (SD) dose of insulin at baseline was 31.98 (11.04) IU/d and 12.51 (5.38) IU/d in the premixed and basal insulin groups, respectively, and was slightly increased to 33.84 (10.97) IU/d and 13.88 (5.84) IU/d in the premixed and basal insulin groups, respectively, at the end of follow‐up (Table S2). The mean (SD) types of concomitant non‐insulin medications used in the basal insulin group were higher than those in the premixed insulin group (Table S2). During the follow‐up period, the percentage of patients who reported a hypoglycemic episode was lower in the basal insulin group than that in the premixed insulin group (9.6% vs 14.9%, *p* = 0.018).

### 3.3 Change in other metabolic parameters

**FIGURE 2:**
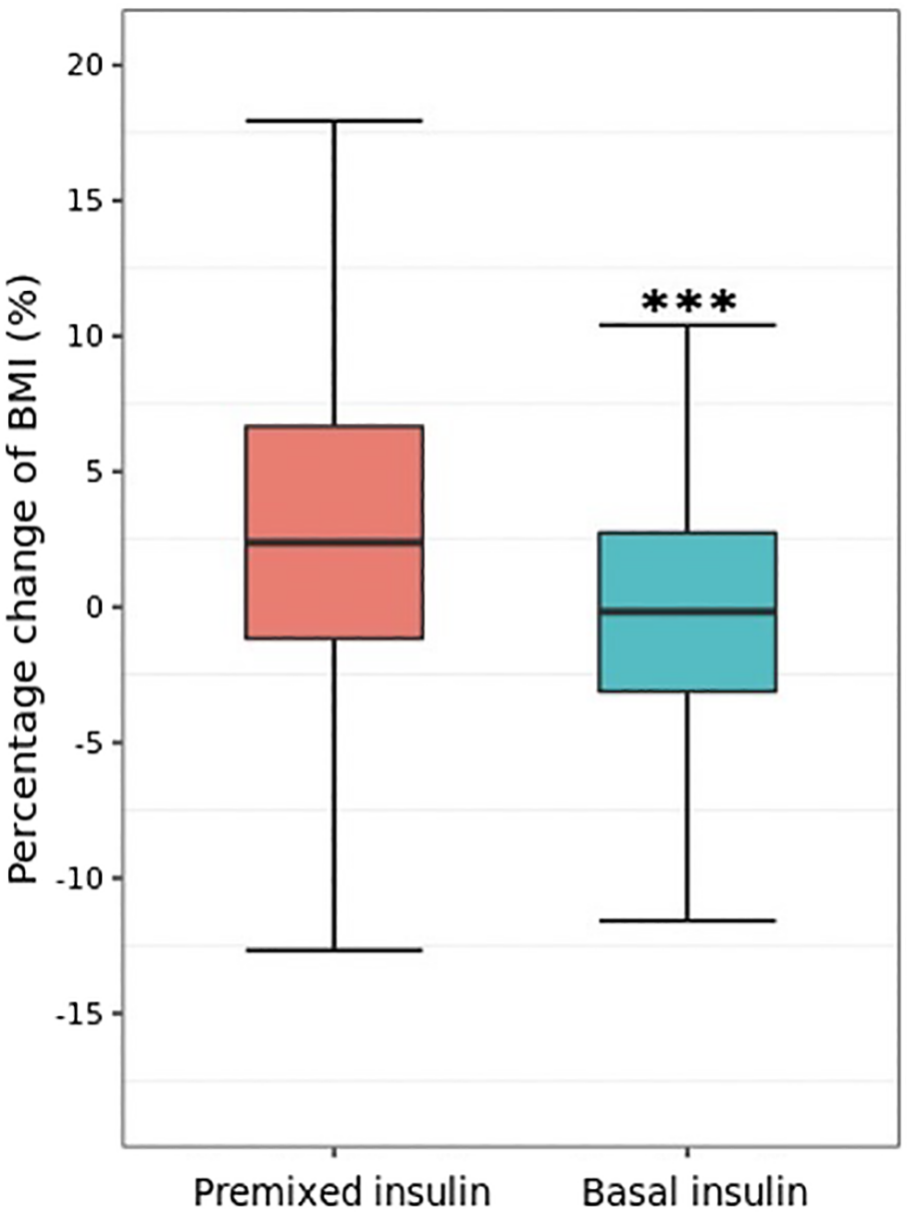
Percentage change of body mass index from baseline. *** p values < 0.0001, refer to comparison between premixed insulin group and basal insulin group using analysis of covariance model adjusted for sex, age and duration of diabetes, BMI, total cholesterol, fasting serum C peptide, and SBP at baseline. BMI, body mass index

In addition to HbA1c, both study groups showed a significant decrease in FBG, total cholesterol, and LDL cholesterol concentrations after treatment (Table 3). The mean (SD) percentage change in BMI from baseline was significantly different between the premixed and basal insulin group (3.40 [6.73]% vs −0.37 [5.50]%, *p* < 0.0001) (Figure 2, Table 3). Similar results were observed in the percentage change of FBG, SBP, DBP, total cholesterol, and triglyceride concentrations between groups (Table 3).

### 3.4 Subgroup analyses

**FIGURE 3:**
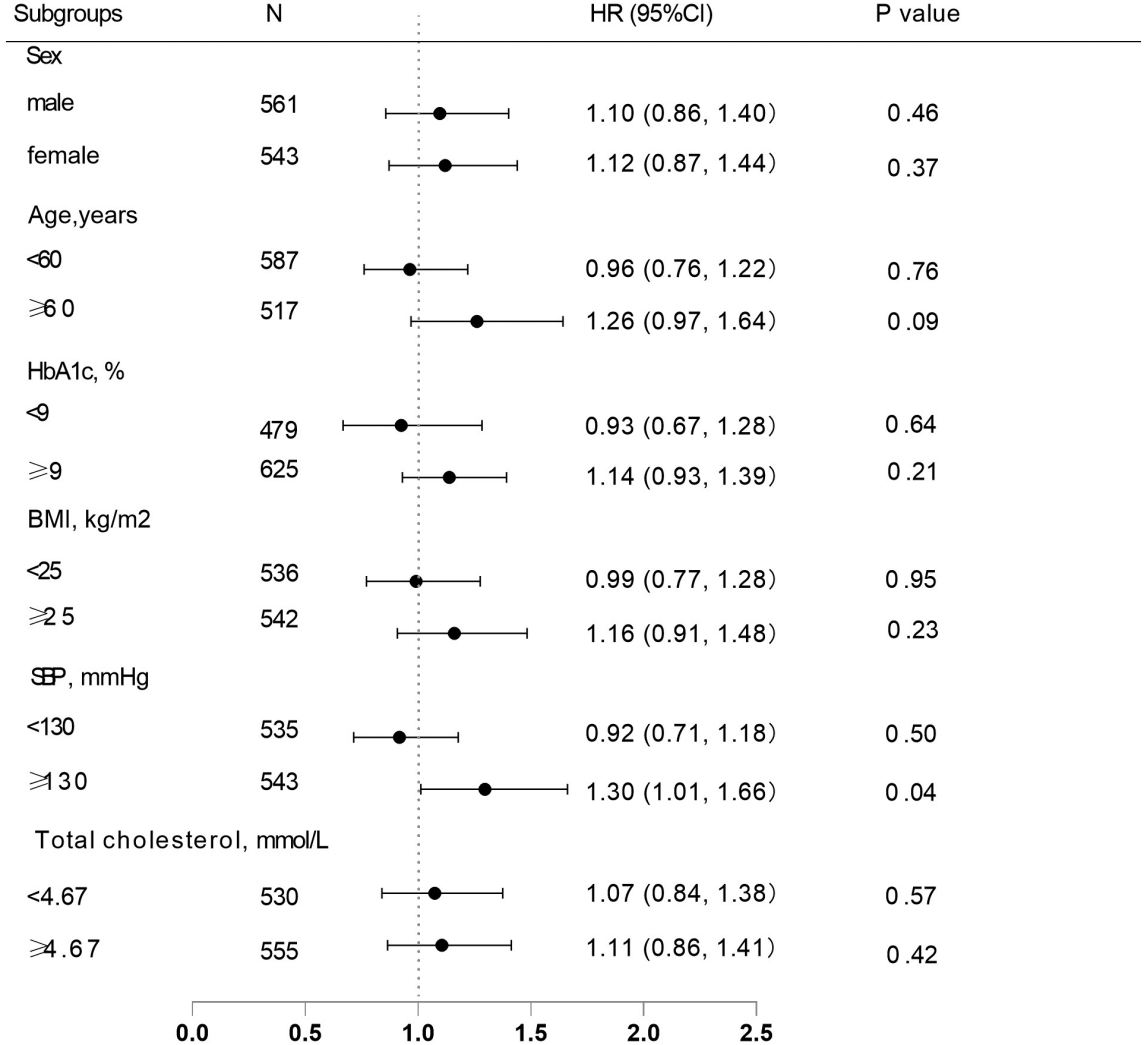
Subgroup analyses on basal and premixed insulin groups reaching glycemic control target. Analyses were adjusting for sex, age, duration of diabetes, total cholesterol, fasting serum C peptide, systolic blood pressure, and HbA1c at baseline, if not be stratified, with premixed insulin as reference group. BMI, body mass index; SBP, systolic blood pressure; DBP, diastolic blood pressure; HbA1c, glycated hemoglobin; CI, confidence interval; HR, hazard ratio

Since bias was observed in certain baseline characteristics in the two insulin treatment groups, we further stratified the participants into subgroups and performed analyses based on multivariable Cox proportional hazards regression models with full adjustment to elucidate the influence of these factors on the efficacy of the two insulin regimens. The results showed that after stratification by sex, age, HbA1c, BMI, and total cholesterol similar results were obtained, and no difference was found in reaching the glycemic control target between the premixed and basal insulin groups. Except that in the stratification with SBP ≥ 130 mmHg a marginally significant difference was found between the two treatment groups (*p* = 0.04) (Figure 3).

## 4 DISCUSSION

In this prospective, large‐scale, real‐world study with a relatively long duration at 10 MMC centers, we investigated the habits of Chinese endocrinologists in insulin prescription and the effects of once‐daily basal insulin and twice‐daily premixed insulin regimens when non‐insulin agents failed to meet glycemic control targets in 1104 T2DM participants. The results indicated that, on the basis of current diabetes guidelines, basal insulin was prescribed more often than premixed insulin at initiation. Patients with a higher BMI and with worse metabolic control were more likely to receive premixed insulin than basal insulin. After a median follow‐up of 12.0 months, treatment with basal insulin showed similar effects on reaching HbA1c targets compared with premixed insulin, even after adjustment and stratification by sex, age, HbA1c, BMI, and total cholesterol, indicating a similar effect on glycemic control between the two insulin regimens.

Once‐daily basal insulin and twice‐daily premixed insulin are two alternatives when initiating insulin therapy in those who failed lifestyle intervention and non‐insulin medications recommended by the Chinese guideline on T2DM management.^9^ This is different from the consensus of ADA and EASD, which only recommended basal insulin administration at this step.^8^, ^10^ Several studies compared the different effects and safety of premixed insulin and basal insulin administration in Chinese patients.^14^, ^15^, ^16^, ^17^, ^18^ One pragmatic real‐world study by Zhang et al indicated that after 24 weeks of treatment least squares mean changes in HbA1c were −2.00% for the basal insulin and −2.15% for the premixed insulin (with mid‐mixture insulin analog) group, with no significant difference found between the two groups. However, the study found that numerically, a higher percentage of patients achieved the HbA1c target in the premixed insulin group (34%) than in the basal insulin group (28.5%). No severe hypoglycemic episode was observed, and both therapies yielded small weight gains during intervention.^15^ Similar results were found by Yang et al., which indicated that a once‐daily premixed insulin analog had a noninferior efficacy on HbA1c reduction in Chinese and Japanese T2DM patients uncontrolled with metformin and a sulfonylurea.^17^ However, a cross‐sectional survey by Liu et al. in 602 Chinese hospitals found a better glycemic control effect in patients receiving premixed insulin compared with those receiving basal insulin, and more patients achieved the HbA1c target than those with basal insulin, even after stratification with different factors.^14^ Similar results were found by Lee et al.^16^ All these studies are highly heterogeneous in design, and no conclusion could be drawn regarding which regimen is better based on this limited evidence.

The present study indicates that patients who failed to meet the HbA1c targets of <7% and switched from baseline non‐insulin medications to either once‐daily basal insulin or twice‐daily premixed insulin regimens both achieved a significant HbA1c decrease after a median follow‐up of 12.0 months. No difference was found in meeting HbA1c targets between the two groups by the Cox proportional hazards regression model adjusting for multiple influencing factors (1.10 [95% CI, 0.92‐1.31; *p* = 0.29]). We chose the time‐to‐HbA1c target as primary outcome since HbA1c < 7% is an important glycemic control target for most of the T2DM patients according to the current guidelines. Furthermore, to achieve a lower HbA1c level might be accompanied with more episodes of hypoglycemia, especially for patients with insulin administration. Therefore, time‐to‐target HbA1c might be a better indicator to reflect the advantages of a certain insulin treatment pattern than a decrease in HbA1c level. Moreover, considering the inconsistency in the baseline HbA1c between the two study groups (participants in the premixed insulin group had a relatively higher baseline HbA1c level than those in the basal insulin group), we added a HbA1c > 10% decrease from baseline as a supplementary criterion in the primary outcome.

Relatively less total daily dosage of insulin but more types of concomitant non‐insulin medications enable the once‐daily basal insulin to achieve similar glycemic control effects compared with twice‐daily premixed insulin. Moreover, less weight gain was observed in those treated with basal insulin than in the premixed insulin group, which might be at least partially attributed to the lower insulin dosage administered in the basal insulin group.

Another important finding in the present study was that at baseline once‐daily basal insulin (57.3%) was more frequently prescribed by doctors than twice‐daily premixed insulin (42.7%). This finding was different from the JADE study which found that during 2007 through 2017 in 11 Asian countries/regions both premixed insulin (44%) and basal‐only (42%) insulin were the two most commonly used insulin regimens with similar percentages of prescriptions.^21^ However, the authors did not offer the change of insulin use pattern over time during such a large time span. Another early study using data from the city of Tianjin during 2009 through 2010 found that while initiating insulin therapy in T2DM patients more patients were prescribed premixed insulin (77.3%) than basal (11.8%) insulin.^22^ These findings, to some extent, reflect the changes and tendency of Chinese doctors during recent years to decrease daily insulin injection frequencies and dosages use in order to increase patient compliance and minimize weight gain when choosing insulin treatment patterns.

Moreover, we investigated the factors that might influence the choice of insulin regimens of the doctors, and we found that the doctors preferred to prescribe twice‐daily premixed insulin (31.98 [11.04] IU/d at initiation) rather than once‐daily basal insulin (12.51 [5.38] IU/d at initiation) to those who had higher BMI and HbA1c levels at initiation. The daily insulin dosages used in the present study in each group were similar to those found in other studies.^15^, ^23^ These findings suggested that doctors preferred the twice‐daily administration of insulin to those with higher insulin dosages used (given on the basis of patient body weight) or with poorer glycemic control at initiation. However, when we further stratified patients into different HbA1c and BMI subgroups, the results showed no difference in the efficacies of glycemic control between the two insulin treatment groups in either the HbA1c < 9% or ≥ 9% subgroups (*p* = 0.64 and 0.21, respectively) or the BMI < 25 or ≥ 25 kg/m^2^ subgroups (*p* = 0.95, and 0.23, respectively). Stratification analyses in other subgroups yielded similar results. Therefore, the results of subgroup analyses indicated that worse metabolic situations, especially HbA1c levels and BMI, might not be considered for the selection of different insulin regimens in those not controlled with non‐insulin medications. Moreover, since less weight gain and fewer hypoglycemic episodes were observed in the basal insulin group than in the premixed insulin group, basal insulin once‐daily accompanied by other non‐insulin medications might be a better choice at this stage for patients who fail to achieve HbA1c targets. However, other factors, including economic affordability, patients' dietary habits, and willingness to accept insulin and non‐insulin combinations and intense visits to the clinics, should also be taken into consideration when changes are made at this stage.^24^

This study has several strengths. First, this is a large, multi‐center, real‐world registry study to investigate insulin administration habits in China in routine clinical practice. Second, the types of insulin used in the present study are varied, which could better reflect the current situation in real‐world practice than a randomized controlled trial (RCT). Moreover, with the relatively longer duration of follow‐up (up to 47 months) rather than cross‐sectional observation, the study results could offer reliable evidence to decision‐makers for future changes to the treatment guidelines in China.

The present study has several limitations. First, because of the prospectively observational study design, the baseline characteristics were not comparable between the two groups, the subgroup analyses were post hoc, and the follow‐up time is limited, which may underestimate the advantages of a certain treatment pattern. However, since real‐world evidence better reflects the proper characteristics in the present situation, the results could be complementary to those from RCTs. Second, the concomitant non‐insulin medications accompanied by insulin therapy were different between participants and study groups; therefore, the comparison of the effects between the two insulin regimens could not be equal to the effects of insulin itself, and we should take caution when interpreting the current findings.

In conclusion, in this real‐world, prospective study with a long follow‐up duration at 10 diabetes centers in China, we investigated the habits of doctors on the choice of insulin initiation patterns when non‐insulin medication failed to meet glycemic control targets. During a median 12.0 months follow‐up, similar glycemic control effects were found between the once‐daily basal insulin and the twice‐daily premixed insulin regimens, with less weight gain and fewer hypoglycemic episodes observed in the basal insulin group, which indicates that the once‐daily basal insulin regimens with concomitant non‐insulin medication might be a preferred treatment pattern when switching to insulin treatment for the failure of non‐insulin medications in Chinese T2DM patients.

## DISCLOSURE

The authors declare no conflicts of interest.

## Supporting information


**Table S1.** Types of hypoglycemic medications involved in the present study
**Table S2.** No. of non‐insulin drugs and daily insulin dosage at baseline and at last visit.
