# Supplementary material for: Effects of basal and premixed insulin on glycemic control in type 2 diabetes patients based on multicenter prospective real‐world data
Source: J Diabetes. 2022 Jan 13;14(2):134–43. doi: 10.1111/1753-0407.13245 (PMC9060040; doi:10.1111/1753-0407.13245)
Supplement: Supplementary file 1 — Table S1. Types of hypoglycemic medications involved in the present study Table S2. No. of non‐insulin drugs and daily insulin dosage at baseline and at last visit. [file JDB-14-134-s001.docx]

**Effects of basal and premixed insulin on glycemic control in type 2 diabetes patients based on multicenter prospective real-world data**

**Supplementary Materials**

**Supplementary Table 1. Types of hypoglycemic medications involved in the present study**

| **Types of hypoglycemic medications** | **No. of participants** | **Percentage of participants (%)** |
| --- | --- | --- |
| **Insulin in both groups** | |  |
| Insulin of animal origin | 1 | 0.1 |
| Human insulin | 181 | 16.4 |
| Insulin analogue | 921 | 83.4 |
| Unknown | 1 | 0.1 |
| **Concomitant non-insulin medications**  **in the premixed insulin group at last visit** | | |
| Metformin | 313 | 66.5 |
| Sulfonylureas | 14 | 3 |
| Alpha glycosidase inhibitors | 167 | 35.5 |
| Thiazolidinediones | 44 | 9.3 |
| Non-sulfonylurea insulin secreting agents | 5 | 1.1 |
| GLP-1 receptor agonist | 8 | 1.7 |
| DPP-4 inhibitor | 71 | 15.1 |
| SGLT2 inhibitor | 116 | 24.6 |
| **Concomitant non-insulin medications**  **in the basal insulin group at last visit** | | |
| Metformin | 460 | 72.7 |
| Sulfonylureas | 137 | 21.6 |
| Alpha glycosidase inhibitors | 346 | 54.7 |
| Thiazolidinediones | 26 | 4.1 |
| Non-sulfonylurea insulin secreting agents | 95 | 15.0 |
| GLP-1 receptor agonist | 47 | 7.4 |
| DPP-4 inhibitor | 194 | 30.6 |
| SGLT2 inhibitor | 228 | 36.0 |

GLP-1, glucagon-like peptide-1; DPP-4, dipeptidyl peptidase-4; SGLT2, sodium-dependent glucose transporters 2.

|  | Total | Premixed insulin | Basal insulin | *P* value*** |
| --- | --- | --- | --- | --- |
| No. of participants | 1,104 | 471 | 633 |  |
| No. of non-insulin drugs at baseline | 2.03 ± 0.93 | 1.62 ± 0.92 | 2.35 ± 0.82 | <0.0001 |
| No. of non-insulin drugs at last visit | 2.06 ± 1.01 | 1.57 ± 0.94 | 2.42 ± 0.89 | <0.0001 |
| Daily insulin dosage at baseline, IU | 20.81 ± 12.70 | 31.98 ± 11.04 | 12.51 ± 5.38 | <0.0001 |
| Daily insulin dosage at last visit, IU | 22.40 ± 12.97 | 33.84 ± 10.97 | 13.88 ± 5.84 | <0.0001 |

**Supplementary Table 2. No. of non-insulin drugs and daily insulin dosage at baseline and at last visit**


**P* values refer to comparison between premixed insulin group and basal insulin group
